# HCA-DAN: hierarchical class-aware domain adaptive network for gastric tumor segmentation in 3D CT images

**DOI:** 10.1186/s40644-024-00711-w

**Published:** 2024-05-21

**Authors:** Ning Yuan, Yongtao Zhang, Kuan Lv, Yiyao Liu, Aocai Yang, Pianpian Hu, Hongwei Yu, Xiaowei Han, Xing Guo, Junfeng Li, Tianfu Wang, Baiying Lei, Guolin Ma

**Affiliations:** 1 Department of Medical Imaging, Heping Hospital Affiliated to Changzhi Medical College, Changzhi, China; 2 School of Biomedical Engineering, Health Science Centers, National-Regional Key Technology Engineering Laboratory for Medical Ultrasound, Marshall Laboratory of Biomedical Engineering, Shenzhen University, Shenzhen, China; 3 Peking University China-Japan Friendship School of Clinical Medicine, Beijing, China; 4 Department of Radiology, China-Japan Friendship Hospital, No. 2 East Yinghua Road, Chaoyang District, Beijing, 100029 China; 5 Department of Radiology, The Affiliated Drum Tower Hospital of Nanjing University Medical School, Nanjing, China; 6 AI Research Center for Medical Image Analysis and Diagnosis, Shenzhen University, Guangdong, China

**Keywords:** Gastric tumor segmentation, Anisotropic network, Domain adaptation, Hierarchical class-aware domain alignment, CT images

## Abstract

**Background:**

Accurate segmentation of gastric tumors from CT scans provides useful image information for guiding the diagnosis and treatment of gastric cancer. However, automated gastric tumor segmentation from 3D CT images faces several challenges. The large variation of anisotropic spatial resolution limits the ability of 3D convolutional neural networks (CNNs) to learn features from different views. The background texture of gastric tumor is complex, and its size, shape and intensity distribution are highly variable, which makes it more difficult for deep learning methods to capture the boundary. In particular, while multi-center datasets increase sample size and representation ability, they suffer from inter-center heterogeneity.

**Methods:**

In this study, we propose a new cross-center 3D tumor segmentation method named Hierarchical Class-Aware Domain Adaptive Network (HCA-DAN), which includes a new 3D neural network that efficiently bridges an Anisotropic neural network and a Transformer (AsTr) for extracting multi-scale context features from the CT images with anisotropic resolution, and a hierarchical class-aware domain alignment (HCADA) module for adaptively aligning multi-scale context features across two domains by integrating a class attention map with class-specific information. We evaluate the proposed method on an in-house CT image dataset collected from four medical centers and validate its segmentation performance in both in-center and cross-center test scenarios.

**Results:**

Our baseline segmentation network (i.e., AsTr) achieves best results compared to other 3D segmentation models, with a mean dice similarity coefficient (DSC) of 59.26%, 55.97%, 48.83% and 67.28% in four in-center test tasks, and with a DSC of 56.42%, 55.94%, 46.54% and 60.62% in four cross-center test tasks. In addition, the proposed cross-center segmentation network (i.e., HCA-DAN) obtains excellent results compared to other unsupervised domain adaptation methods, with a DSC of 58.36%, 56.72%, 49.25%, and 62.20% in four cross-center test tasks.

**Conclusions:**

Comprehensive experimental results demonstrate that the proposed method outperforms compared methods on this multi-center database and is promising for routine clinical workflows.

## Introduction

Image-guided disease diagnosis and treatment is an important part of routine clinical workflow, particularly for gastric cancer, which is the third leading cause of cancer-related death worldwide [1]. Computed tomography (CT) is the most commonly used imaging modality for preoperative assessment of tumor status, because it has the advantages of high imaging density resolution, convenient inspection, fast acquisition speed, and non-invasiveness [2]. In clinical practice, imaging examination is usually performed manually by radiologists slice by slice [3], which is an expensive and time-consuming process and also relies heavily on the experience of radiologists. Automated segmentation of gastric tumors not only reduces the burden of radiologists, but also is expected to supplement the conventional imaging tools. However, this segmentation task is challenging due to the following reasons: (a) there exist anisotropic spatial resolution in 3D CT images, (b) low contrast between tumor and adjacent structures, (c) the large samples needed to train robust models are often difficult to obtain from a single medical center.

Previous studies using CT images to characterize gastric cancer were mainly oriented to some diagnostic tasks (e.g., estimate tumor invasion depth, predict lymph node metastasis, and identify occult peritoneal metastasis, etc.) [4–7], and these works usually performed task-specific predictions based on the region of interest (ROI) of the primary tumor. In previous work, computer-aided diagnosis (CAD) methods are mainly based on radiomics to study gastric cancer in CT images. For example, Wang et al. [8] explored the potential performance of radiomics-based method for predicting the depth of tumor invasion in gastric cancer by performing tumor segmentation using dedicated post-processing software from enhanced CT images. Meng et al. [9]. extracted 2D and 3D CT radiomic features from multi-center dataset and comprehensively compared 2D and 3D radiomic features for gastric cancer characterization and discrimination in three diagnostic tasks. Dong et al. [10]. identified occult peritoneal metastasis in 554 gastric cancer patients from four centers. They first build radiomic signatures of the primary tumor and peritoneum based on 266 imaging features, and then combined the primary tumor, peritoneum and the Lauren types to predict the occult peritoneal metastasis. The above studies are all based on radiomics, which usually includes two stages: extracting ROI-based hand-crafted features and building traditional machine learning classifiers. The extraction of radiomic features is a very time-consuming feature engineering that usually requires domain-specific expertise. Furthermore, the methods proposed in the above works are not fully automatic and are not suitable for studying multi-center data due to complex data distribution and huge feature engineering.

With the rapid development of deep learning technology, CAD algorithms based on deep learning have achieved convincing performance in medical image analysis [11–13], particularly in some abdominal CT image analysis [14–17]. Previous CNN-based deep learning methods were inevitably limited in modeling long-term dependencies by ignoring non-local correlations of images. Inspired by the success of Transformers in natural language processing (NLP) and computer vision (CV), Transformers is being widely used in medical image processing [18–20] as an alternative backbone for CNNs due to its ability to capture long-term dependencies. However, only a few deep learning-based CAD algorithms [21–23] have been proposed for automatic segmentation of gastric tumors from CT images, and these works are from us. In [21–23], we collected data from three medical centers to increase the sample size, but ignored the heterogeneity/shift of data from different sources. In medical image analysis, domain heterogeneity/shift is more prominent than conventional common data due to the changes of scanning instruments and the diversity of hospital population. Domain adaptation techniques are designed to reduce the domain shift and make the model go towards better generalization in the test phase. When the data distribution gap between source and target domains is narrowed, an improved generality can be obtained. For unsupervised domain adaptation (UDA), a specific scenario is when we have data from two or more medical centers/sites, it is usually assumed that the unlabeled data of one of the medical centers is the target domain, and the labeled data of the remaining one or more medical centers is source domain [24]. UDA algorithms narrow domain discrepancy by strengthening information alignment from the perspectives of feature-level or image-level, and then improve the models performance with unlabeled target domain. Feature-level alignment-based methods transform source and target data into latent spaces, aiming to discover domain-invariant features by performing distributional alignment. Most of the methods adopt a Siamese architecture similar to the domain adversarial neural network (DANN) structure [25], which helps to obtain domain-invariant features. Image-level alignment-based methods are often used on paired data, which convert source images into target-like images and vice versa, facilitating segmentation models to learn specific information in the target domain. For example, Zhang et al. [26]. proposed a DANN-based domain-symmetric networks to achieve feature distribution invariance at a finer category level. The proposed network is a symmetric design for source task and target task classifiers, and on this basis, the authors also build an additional classifier that shares neurons with the task classifiers. Hoffman et al. [27]. proposed a model named Cycle-Consistent Adversarial Domain Adaptation (CyCADA), which adapts between domains using both generative image space alignment and latent representation space alignment. Inspired by the above work, some studies have investigated domain adaptation of deep neural networks and applied them to medical image analysis tasks. For example, Kamnitsas et al. [28]. developed an unsupervised domain adaptation method for brain lesion segmentation by investigating adaptation between databases acquired using two different scanners with difference MR imaging sequences. Yan et al. [29]. proposed an adversarial learning based UDA method for cross-vendor medical image segmentation. A domain discriminator is co-trained with the segmentor to learn domain-invariant features for the task of segmentation. Panfilov et al. [30]. developed an unsupervised domain adaptive segmentation model based on adversarial learning for cross-device knee tissue segmentation. A U-Net-based segmentor and a domain discriminator with adversarial learning are co-trained for UDA. However, these above methods ignore class information in feature alignment, which results in misalignment.

In this paper, we propose a new hierarchical class-aware domain adaptive network (HCA-DAN) for gastric tumor segmentation in cross-center scenario. To simultaneously deal with anisotropy in 3D data and the long-range dependency on the extracted feature maps, we design a feature extraction backbone that efficiently bridges an Anisotropic neural network and a Transformer (AsTr) for extracting multi-scale features from the CT images. In particular, we also design a pyramid boundary-aware (PBA) block that is placed at multiple levels in the decoding path. Furthermore, we propose a hierarchical class-aware domain alignment (HCADA) module, which not only considers tumor size in feature alignment, but also incorporates a class attention map into the domain discriminator to make the feature alignment process pay more attention to the class-specific information. In summary, our work has three main contributions:

- We develop a new unsupervised domain adaptive framework, which can not only learn discriminative multi-scale features, but also narrow domain heterogeneity/shift between cross-center datasets.
- A new feature extraction backbone AsTr, is designed, which not only considers the anisotropy of 3D volume, but also alleviates the shortcomings of CNNs in modeling long-term dependence. Furthermore, the PBA block is aggregated into the decoding path to enhance the ability of AsTr to capture the boundaries of tumors.
- A new domain adaptive module HCADA, is proposed, which guides the network to capture class-specific rather than class-agnostic knowledge for multi-scale feature distribution alignment.

## Materials and methods

### Datasets and data pre-processing

This is a retrospective multi-center study with data from the four medical centers (Taiyuan People Hospital, China; Xian People Hospital, China; Department of Radiology, China-Japan Friendship Hospital, Beijing, China; Heping Hospital, Changzhi Medical College, China) by four kinds of medical instruments (Toshiba320-slice CT, SOMATOM 64-slice CT, Philips 128-slice CT and SOMATOM force dual source CT), with a largely varying in-plane resolution from 0.5 mm to 1.0 mm and slice spacing from 5.0 mm to 8.0 mm. For simplicity, we represent the above four datasets as D1, D2, D3 and D4, respectively. Our dataset was collected from 2015 to 2018, which contains 211 CT image samples (211 ordinary CT volumes and 63 enhanced CT volumes), of which D1 included 74 cases, D2 included 39 cases, D3 included 47 cases (47 ordinary CT volumes and 63 enhanced CT volumes), and D4 included 51 cases. The ground truth of segmentation is annotated by four experienced radiologists using the ITK-SNAP software based on surgical pathology. The four experienced radiologists all specialize in abdominal radiology, two of them have 8 years of clinical experience and the other two have more than 10 years of clinical experience. Note that the used dataset has passed the ethical review of the relevant hospitals and obtained the informed consent of the patients.

To cope with the limitation of 3D data on computer memory consumption, and considering that the tumor area is smaller than the background area, we cut and resample each volume to patches including voxels with a voxel size of 5.0 × 0.741 × 0.741mm^3^ or 8.0 × 0.741 × 0.741 mm^3^. To compensate for the lack of training data, we not only use the online data augmentation [12] (e.g., flipping, rotation, translation), but also perform CT image normalization (automatic clipping operation from 0.5 to 99.5% intensity value of all foreground voxels) and voxel space resampling (with third order spline interpolation).

### Network overview

**Fig. 1:**
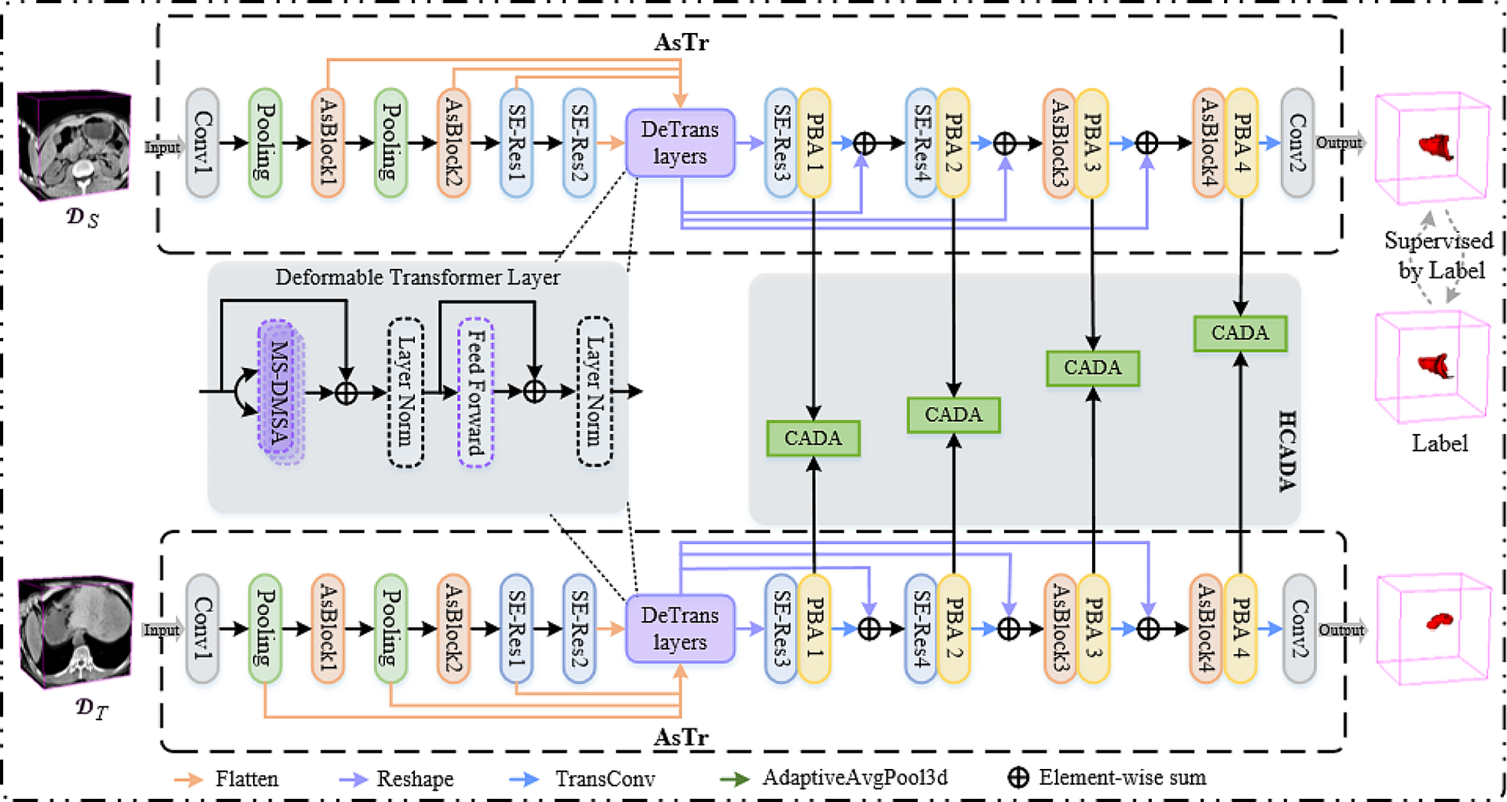
The overview of the proposed HCA-DAN. AsBlock: anisotropic convolutional block; SE-Res: squeeze-and-excitation residual block; PBA: pyramid boundary-aware block; HCADA: hierarchical class-aware domain alignment module, which includes four CADA blocks. Note that to demonstrate an elegant framework, we omit the display of the positional encoding when the multi-scale features generated from the As-encoder are passed to the DeTrans layer

Figure 1 shows the overview of the proposed HCA-DAN, which includes two collaborative components, i.e., AsTr and HCADA. The proposed 3D domain adaptation network takes an abdominal CT volume as input and starts with AsTr as backbone to extract multi-scale context features from the CT images with anisotropic resolution. Then the extracted features from source and target domains are passed to HCADA module, which can effectively distinguish the features of the source and target domains by taking into account class information.

### Architecture of AsTr

Inspired by CoTr [18], AsTr is proposed to learn more discriminative multi-scale features for gastric tumor segmentation via jointing CNN and Transformer. AsTr consists of an anisotropic convolutional encoder (As-encoder) for feature extraction from the CT images with anisotropic resolution, a deformable Transformer-encoder (i.e., DeTrans-encoder) for long-range dependency modeling, an anisotropic convolutional decoder (As-decoder) for accurate tumor segmentation.

**Fig. 2:**
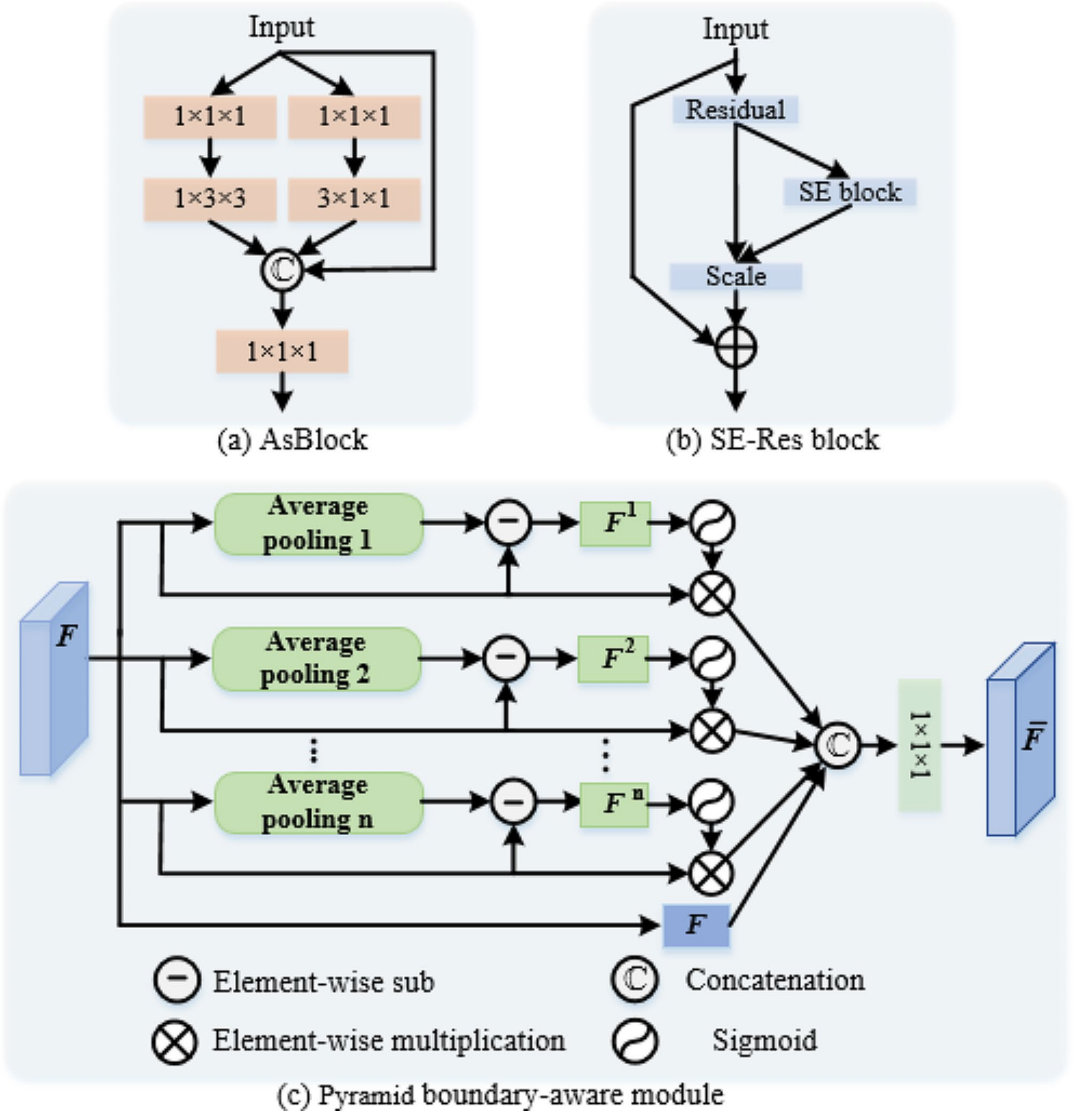
The architectures of three blocks

To address the issue of anisotropic voxel resolution, we construct the As-encoder by combining anisotropic convolution with isotropic convolution, rather than simply using isotropic convolution. The As-encoder mainly contains a Conv-GN-PReLU block, two average pooling layers, two stages of anisotropic convolution block (AsBlock), and two stages of 3D squeeze-and-excitation residual (SE-Res) block. The Conv-GN-PReLU block represents a 3D convolutional layer followed by a group normalization (GN) and a parametric rectified linear unit (PReLU). The number of AsBlock in two stages are two and three, respectively. The number of SE-Res block in two stages are three and two, respectively. As shown in Fig. 2a, the input of AsBlock is delivered to 1 × 3 × 3 and 3 × 1 × 1 anisotropic convolutions, respectively. Then the outcomes are then concatenated with the input as the output. Moreover, the 1 × 1 × 1 convolution are employed to both input and output to adjust the channel numbers of features. Through this design, the As-encoder can independently extract features on the x-y plane and along the z direction from 3D volume, which reduces the influence of anisotropic spatial resolution. Considering that 3D data contains a wealth of information, we add two stages of SE-Res block in the back end of the As-encoder. As shown in Fig. 2b, the SE-Res block consists of residual and SE blocks, which not only improves the representation capability of the encoder, but also alleviates the overfitting problem caused by the deep network.

To compensate for the inherent locality of convolution operation, the DeTrans layer is proposed [18] to capture the long-term dependence of pixels in multi-scale features generated by the encoder. In general, the DeTrans layer is composed of a multi-scale deformable self-attention (MS-DMSA) layer and a feedforward network, each being followed by the layer normalization.

To capture more accurate tumor boundaries, in addition to AsBlock and SE-Res blocks, we also design the PBA block in As-decoder. Therefore, the As-decoder mainly contains two stages of AsBlock, two stages of 3D SE-Res block, four PBA blocks, four transpose convolution layers, and a Conv-GN-PReLU block. Inspired by 2D pyramid edge extraction module [31], we design the 3D PBA block (as shown in Fig. 2c) to refine the boundaries of the lesion. The PBA block is a simple and effective pyramid boundary information extraction strategy, which can obtain robust boundary information by capturing different representations of pixels around the diseased area. Specifically, the PBA block takes the features ***F*** generated by the previous layer as input and passes it into a multi-branch pooling layer with different kernel sizes to obtain the features $\left\{{\varvec{F}}^{1},\cdots ,{\varvec{F}}^{\text{n}}\right\}$ with lesion edge information. Then, the feature $\stackrel{-}{\varvec{F}}$ is generated by a series of operations, which can be defined as:

1 $$\overline{\boldsymbol{F}}=\operatorname{conv}\left\{\mathbb{C}\left[\sigma\left(\boldsymbol{F}-\boldsymbol{F}^1\right) \otimes \boldsymbol{F} ; \cdots ; \sigma\left(\boldsymbol{F}-\boldsymbol{F}^{\mathrm{n}}\right) \otimes \boldsymbol{F}\right]\right\}$$

where $\left\{{\varvec{F}}^{1},\cdots ,{\varvec{F}}^{\text{n}}\right\}$ is obtained by average pooling layers with different kernel sizes; conv means a 1 × 1 × 1 convolutional layer; $\mathbb{C}$ represents channel concatenation operation; $\sigma$ denotes a Sigmoid function; $\otimes$ indicates element-by-element multiplication. In this way, we obtain multiple granularities responses near the edge by subtracting the value of average pooling with different sizes from its local convolutional feature maps and configuring soft attention operation in each branch.

It is worth noting that during decoding, the output sequence of the DeTrans layers is reshaped into feature maps according to the size at each scale. Then, the reshaped multi-scale features are added element-by-element in the decoding path for better tumor segmentation.

### Hierarchical class-aware domain alignment

In this section, we consider how to use the class-specific information to guide multi-scale feature distribution alignment in our feature extractor AsTr. On the one hand, tumors of different cases have different sizes and positions in CT images, and multi-scale feature extraction has been proved to be very effective in many scenarios, especially in the task of lesion segmentation. Technically, low resolution feature maps tend to predict large objects, while high resolution feature maps tend to predict small objects. Therefore, we introduce the hierarchical domain alignment mechanism, which takes object scales roughly into account when performing domain distribution alignment. In short, we configure a domain discriminator for each scale feature, which can effectively guide the feature alignment of tumors of different sizes. On the other hand, many efforts ignore class-specific knowledge during feature alignment, which leads to misalignment. To encourage a more discriminative distribution alignment, we produce an attention map for each class separately, which is calculated based on the probability of class occurrence. The attention map is defined as:

2 $${\varvec{F}}_{att}=\text{S}\text{o}\text{f}\text{t}\text{m}\text{a}\text{x}\left({\varvec{F}}_{out}\right)$$,

where ${\varvec{F}}_{out}$ denotes the output of the segmentation network AsTr. In other words, we use the $\text{S}\text{o}\text{f}\text{t}\text{m}\text{a}\text{x}$ function to calculate the class attention map for all output spatial positions. This class attention map is aggregated into the domain discriminator to capture class-specific information in domain adaptation, rather than class-agnostic information, which encourages more discriminative distribution alignment in the CADA block. Specifically, we employ the U-Net [32] architecture as a domain discriminator *D* in the CADA block. First, we upsample the feature generated by the PBA block with triple interpolation to the same resolution as the input image. The newly generated feature is then fed into the domain discriminator *D* and a probability map is generated to distinguish whether the feature is from the source or target domains. Finally, this probability map is multiplied by the class attention map element by element to obtain the final probability map.

### Data partitioning and network implementation

We validate the proposed method in both in-center and cross-center test scenarios. In order to obtain reliable segmentation results, we employed a five-fold group cross-validation strategy in the in-center test scenario. In the cross-center test scenario, we use three datasets as the source domain and the remaining one as the target domain, which is a common validation strategy for domain adaptive methods.

The proposed cross-center 3D tumor segmentation method is implemented on the PyTorch platform and is trained with 1x NVIDIA GeForce RTX 3090 GPU (24GB). We train all 3D networks by using the SGD optimizer with a momentum of 0.99 and an initial learning rate of 1 × 10^− 3^. We set batch size as 2, and the network was trained for 500 epochs and each epoch contains 250 iterations. In four PBA blcoks, we use the 3 × 3 × 3 and 5 × 5 × 5 average pooling operation for the first two blocks, and 5 × 5 × 5 and 7 × 7 × 7 pooling kernels in the last two blocks.

We employ four performance metrics to quantitatively evaluate the obtained segmentation results, which include the Dice similarity coefficient (DSC), Jaccard index (JI), Average surface distance (ASD, in mm) and 95% Hausdorff distance (95HD, in mm). The first two are more sensitive to the inner filling of the mask, and the second two are more sensitive to the segmentation boundary. These metrics are calculated by the following formulas:

3 $$DSC=\frac{2\left|prediction\bigcap groundtruth\right|}{\left|prediction\right|+\left|groundtruth\right|}$$

4 $$JI=\frac{\left|prediction\bigcap groundtruth\right|}{\left|prediction\right|+\left|groundtruth\right|-\left|prediction\bigcap groundtruth\right|}$$

5 $$ASD=\frac{1}{2}\left\{{mean}_{x\in X} {min}_{y\in Y} d\left(x,y\right), {mean}_{y\in Y} {min}_{x\in X} d\left(x,y\right)\right\}$$

6 $$HD=max\left\{{max}_{x\in X} {min}_{y\in Y} d\left(x,y\right), {max}_{y\in Y} {min}_{x\in X} d\left(x,y\right)\right\}$$

where $\left|*\right|$ and $\cap$ denote the size and the intersection operation in the set, respectively. *x* and *y* are the coordinates of the midpoint of the image, ${mean}_{x\in X} {min}_{y\in Y}$ is average of the closest distance between two points, ${max}_{x\in X} {min}_{y\in Y}$ is the shortest distance from a point in a point set to another point set. 95% HD is similar to maximum HD, which is based on the 95th percentile of the distance between the boundary points in *X* and *Y*.

### Loss function

We employ adversarial strategies to implement network training. Therefore, the proposed network consists of three losses, including segmentation loss ${\mathcal{L}}_{seg}$, discrimination loss ${\mathcal{L}}_{dis}^{h}$ and adversarial domain adaptation loss ${\mathcal{L}}_{da}^{h}$. The segmentation loss is the sum of Dice loss ${\mathcal{L}}_{dice}$ and binary cross-entropy loss ${\mathcal{L}}_{bce}$, which defined as:

7 $${\mathcal{L}}_{seg}={\mathcal{L}}_{dice}+{\mathcal{L}}_{bce}$$

8 $${\mathcal{L}}_{dice}=1-\frac{2{\sum }_{i=1}^{N}{p}_{i}{g}_{i}}{{\sum }_{i=1}^{N}{{p}_{i}}^{2}+{\sum }_{i=1}^{N}{{g}_{i}}^{2}}$$

9 $${\mathcal{L}}_{bce}=\sum _{i=1}^{N}{g}_{i}\text{log}{p}_{i}+\sum _{i=1}^{N}{(1-g}_{i})\text{log}(1-{p}_{i})$$

where *N* is the voxel number of the input CT volume; ${p}_{i}\in \left[\text{0.0,1.0}\right]$represents the voxel value of the predicted probabilities; ${g}_{i} \in \left\{\text{0,1}\right\}$ denotes the voxel value of the binary ground truth volume.

Following [33], we calculate the single-level discrimination and adversarial domain adaptation losses with the least squares loss function as follows:

10 $${\mathcal{L}}_{dis}^{l}={\left[D\left({f}_{PBA}^{l}\left({\varvec{F}}_{\text{s}}^{l}\right)\right)-1\right]}^{2}+{\left[D\left({f}_{PBA}^{l}\left({\varvec{F}}_{\text{t}}^{l}\right)\right)+1\right]}^{2}$$

11 $$\mathcal{L}_{d a}^l=\boldsymbol{F}_{a t t} \otimes\left[D\left(f_{P B A}^l\left(\boldsymbol{F}_{\mathrm{t}}^l\right)\right)-1\right]^2$$

where ${f}_{PBA}^{l}$ denotes *l*-th PBA block; $l\in \{1, 2, 3, 4\}$; ${\varvec{F}}_{\text{s}}^{l}$ and ${\varvec{F}}_{\text{t}}^{l}$ represent the source domain and target domain features obtained in the layer before the *l*-th PBA block, respectively. Therefore, the hierarchical discrimination and adversarial domain adaptation losses are defined as:

12 $${\mathcal{L}}_{dis}^{h}={\sum }_{l=1}^{l=4}{{\lambda }^{l}\cdot \mathcal{L}}_{dis}^{l}$$

13 $${\mathcal{L}}_{da}^{h}={\sum }_{l=1}^{l=4}{{\lambda }^{l}\cdot \mathcal{L}}_{da}^{l}$$

where ${\lambda }^{l}$ denotes the weight of *l*-th discrimination and adversarial domain adaptation losses, which decreases exponentially with the decrease of feature resolution.

## Results

### Comparison with the state-of-the-art segmentation methods

To confirm the efficacy of the proposed AsTr, we compared it with six baseline/state-of-the-art (SOTA) medical image segmentation methods, including V-Net [34], 3D FPN [35], nnU-Net [12], CoTr [18], UNETR [19], and Swin-Unet [20]. V-Net is designed to solve the 3D volume segmentation and is widely used in the segmentation task based on 3D medical image data. 3D FPN is an effective method to extract multi-scale features, and it is used as a backbone for feature extraction in many works. nnU-Net is a robust segmentation method, which has achieved good results in many medical image segmentation tasks. CoTr is an efficient and effective method to bridge CNN and Transformer for 3D medical image segmentation. UNETR consists of a transformer encoder that directly utilizes 3D patches and is connected to a CNN-based decoder via skip connection. Swin-Unet is a pure Transformer-based U-shaped Encoder-Decoder network. We compare the first four methods in the in-center test scenario, and compare all methods in the cross-center test scenario. Tables 1 and 2 list the segmentation results of the above methods and the proposed method in in-center test and cross-center test scenarios, respectively. Compared with other segmentation networks, the proposed AsTr achieves the best segmentation performance and proves that it is an effective method for medical image segmentation by considering data anisotropy in the encoder and decoder. Specifically, our AsTr yields the mean DSC value of 59.26%, 55.97, 48.83%, and 67.28% in four in-center test scenarios, respectively. Compared with other segmentation methods (i.e., V-Net, 3D FPN, nnUNet, and CoTr), our method increases DSC by (17.16%/19.81%/1.33%/15.00%, 19.03%/15.16%/11.94%/10.79%, 24.22%/16.48%/2.41%/13.22%, 12.77%/9.38%/0.88%/9.83%) in four in-center test scenarios, respectively. In addition, our AsTr yields the DSC value of 60.62%, 46.54%, 55.94%, and 56.42% in four cross-center test scenarios, respectively. Compared with other segmentation methods (i.e., V-Net, 3D FPN, nnUNet, UNETR, Swin-Unet and CoTr), our method increases DSC by (18.82%/15.55%/9.24%/10.86%/7.44%/7.71%, 31.21%/16.76%/6.69%/5.49%/2.67%/6.62%, 28.16%/30.85%/15.77%/19.40%/18.39%/14.42%, 7.77%/5.12%/1.66%/5.70%/1.31%/2.62%) in four cross-center test scenarios, respectively. To quantitatively analyze the gain significance of the proposed method compared to other methods, we employ the paired t-test to calculate *p*-value representing comparative significance. As shown in Table 3, our method is well ahead of these baseline methods in four in-center test scenarios (*p*<0.05), where significantly outperforming nnUNet in internal validation for D2 and D3, and rivaling nnUNet in internal validation for D1 and D4.

**Table 1:** Segmentation results of different methods in the in-center test scenario

Test	Method	DSC (%) $\uparrow$	JI (%) $\uparrow$	ASD$\downarrow$	95HD$\downarrow$
D1	V-Net	50.58 ± 4.53	37.12 ± 3.65	12.94 ± 1.76	24.87 ± 5.58
	3D FPN	49.46 ± 2.30	36.76 ± 2.17	13.29 ± 2.25	26.19 ± 5.57
	nnU-Net	58.48 ± 4.50	44.13 ± 3.70	**4.52 ± 2.18**	**16.01 ± 1.33**
	CoTr	51.53 ± 7.81	37.36 ± 6.96	12.55 ± 7.77	26.08 ± 6.42
	**AsTr (Ours)**	**59.26 ± 4.01**	**45.16 ± 3.56**	4.84 ± 2.52	17.04 ± 1.81
D2	V-Net	47.02 ± 2.87	33.31 ± 2.47	12.72 ± 2.49	27.82 ± 2.45
	3D FPN	48.60 ± 3.49	34.92 ± 2.57	14.34 ± 3.11	29.49 ± 6.62
	nnU-Net	50.00 ± 6.72	35.77 ± 5.18	14.36 ± 11.89	32.43 ± 12.36
	CoTr	50.52 ± 3.79	36.13 ± 3.64	8.69 ± 5.63	27.20 ± 8.33
	**AsTr (Ours)**	**55.97 ± 3.67**	**41.13 ± 2.52**	**8.24 ± 5.55**	**22.50 ± 6.55**
D3	V-Net	39.31 ± 8.38	27.27 ± 7.73	16.61 ± 4.05	38.40 ± 8.43
	3D FPN	41.92 ± 6.73	30.07 ± 5.84	20.84 ± 5.13	33.81 ± 9.21
	nnU-Net	47.68 ± 4.80	34.52 ± 4.52	14.58 ± 6.80	30.40 ± 5.50
	CoTr	43.13 ± 6.79	30.06 ± 5.81	14.21 ± 5.24	35.48 ± 8.69
	**AsTr (Ours)**	**48.83 ± 4.50**	**35.15 ± 4.10**	**10.52 ± 4.56**	**27.06 ± 3.92**
D4	V-Net	59.66 ± 7.30	44.44 ± 6.52	7.35 ± 6.20	26.32 ± 11.50
	3D FPN	61.51 ± 6.73	47.54 ± 6.21	7.69 ± 5.87	24.42 ± 9.42
	nnU-Net	66.69 ± 6.75	51.91 ± 6.67	3.92 ± 1.84	18.10 ± 3.23
	CoTr	61.26 ± 4.46	46.66 ± 3.43	7.01 ± 7.88	26.58 ± 9.99
	**AsTr (Ours)**	**67.28 ± 7.63**	**52.81 ± 8.41**	**3.19 ± 0.98**	**17.02 ± 6.03**

**Table 2:** The *p*-values of the paired t-test between the proposed AsTr and other methods in terms of DSC.

Test	V-Net vs. AsTr	3D FPN vs. AsTr	nnUNet vs. AsTr	CoTr vs. AsTr	
D1	0.006	0.005	0.588	0.039	
D2	0.007	0.017	0.016	0.010	
D3	0.016	0.014	0.045	0.026	
D4	0.019	0.027	0.698	0.039	

**Table 3:** Segmentation results of different methods in the cross-center test scenario

Training	Test	Method	DSC	JI	ASD	95HD
{D1, D2, D3}	D4	V-Net	51.02	36.64	12.03	35.21
		3D FPN	52.46	38.05	13.27	30.60
		nnU-Net	55.49	40.28	9.42	29.58
		UNETR	54.68	39.52	12.35	33.71
		Swin-Unet	56.42	41.76	9.26	27.28
		CoTr	56.28	41.54	8.71	26.17
		**AsTr (Ours)**	**60.62**	**46.88**	**8.13**	**25.80**
{D1, D2, D4}	D3	V-Net	35.47	24.34	15.47	40.04
		3D FPN	39.86	28.75	14.62	40.19
		nnU-Net	43.62	31.18	13.71	39.41
		UNETR	44.12	31.71	13.92	38.75
		Swin-Unet	45.33	33.06	11.78	35.62
		CoTr	43.65	31.19	13.33	39.57
		**AsTr (Ours)**	**46.54**	**33.61**	**10.97**	**30.92**
{D1, D3, D4}	D2	V-Net	43.65	30.82	18.95	38.67
		3D FPN	42.75	29.78	20.32	39.41
		nnU-Net	48.32	34.79	15.31	33.01
		UNETR	46.85	33.12	18.34	36.98
		Swin-Unet	47.25	33.60	16.54	36.52
		CoTr	48.89	35.35	14.89	31.60
		AsTr (Ours)	**55.94**	**42.15**	**10.50**	**24.52**
{D2, D3, D4}	D1	V-Net	52.35	38.96	12.81	28.69
		3D FPN	53.67	40.22	10.60	25.96
		nnU-Net	55.50	42.30	9.52	24.20
		UNETR	53.58	40.15	11.62	27.38
		Swin-Unet	55.69	42.51	10.98	24.5
		CoTr	54.98	41.16	**7.41**	**20.76**
		**AsTr (Ours)**	**56.42**	**43.14**	8.68	23.29

To confirm the efficacy of the proposed HCA-DAN, we compared it with three feature-level domain alignment methods, including Kamnitsas et al. [32], Yan et al. [33]. and Panfilov et al. [34]. For simplicity, we named the above methods UDA1, UDA2, and UDA3, respectively. These methods are similar to the proposed HCA-DAN in that they train a segmenter and one/more domain discriminators in an end-to-end manner. Table 4 list the segmentation results of different UDA methods and the proposed method in the cross-center test scenario. The proposed HCA-DAN achieves the best segmentation results, indicating that the segmentation performance of AsTr could be improved by considering both tumor size and class-specific information in the feature alignment process. Compared with AsTr, the DSC values increased by 2.61%, 5.82%, 1.39% and 3.44% in the four cross-center test tasks, respectively.

**Table 4:** Segmentation results of different UDA methods in the cross-center test scenario

Training	Test	Method	DSC	JI	ASD	95HD
{D1, D2, D3}	D4	AsTr (Ours)	60.62	46.88	8.13	25.80
		UDA1	59.17	45.36	8.65	26.31
		UDA2	59.80	46.01	8.42	24.95
		UDA3	61.45	47.32	8.97	25.46
		**HCA-DAN (Ours)**	**62.20**	**47.98**	**7.55**	**22.63**
{D1, D2, D4}	D3	AsTr (Ours)	46.54	33.61	10.97	**30.92**
		UDA1	44.95	32.06	13.17	35.60
		UDA2	45.52	32.75	12.50	33.26
		UDA3	47.96	35.58	11.42	34.67
		**HCA-DAN (Ours)**	**49.25**	**36.86**	**10.87**	31.68
{D1, D3, D4}	D2	AsTr (Ours)	55.94	42.15	**10.50**	**24.52**
		UDA1	54.63	40.91	12.88	29.50
		UDA2	53.86	39.99	15.80	32.49
		UDA3	55.38	41.97	12.42	28.72
		**HCA-DAN (Ours)**	**56.72**	**43.39**	11.05	26.39
{D2, D3, D4}	D1	AsTr (Ours)	56.42	43.14	8.68	23.29
		UDA1	55.95	42.61	9.15	26.33
		UDA2	56.87	43.63	7.32	21.75
		UDA3	56.24	42.97	8.20	25.41
		**HCA-DAN (Ours)**	**58.36**	**45.18**	**6.91**	**20.64**

### Ablation study

To demonstrate the effectiveness of the proposed method for gastric tumor segmentation, we conducted two groups of ablation experiments.

#### Effectiveness of the PBA block

**Fig. 3:**
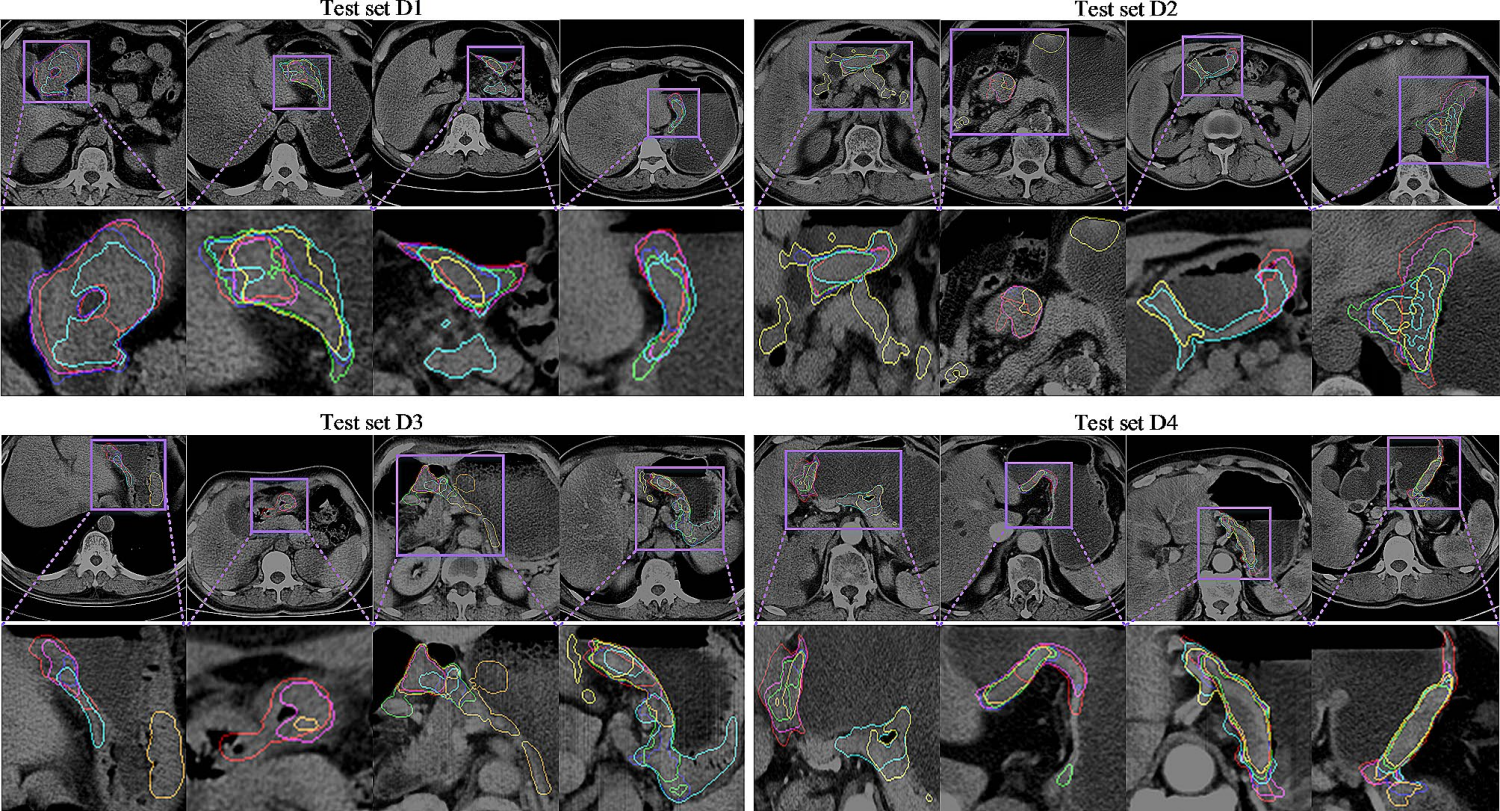
2D visual segmentation boundary comparison of different segmentation networks. The lines represent the true lesion boundaries or predicted boundaries. Ground truth (red) and corresponding tumor boundaries using V-Net (yellow), 3D FPN (cyan), nnU-Net (lime), CoTr (blue), AsTr with (fuchsia) or without (orange) PBA block

**Fig. 4:**
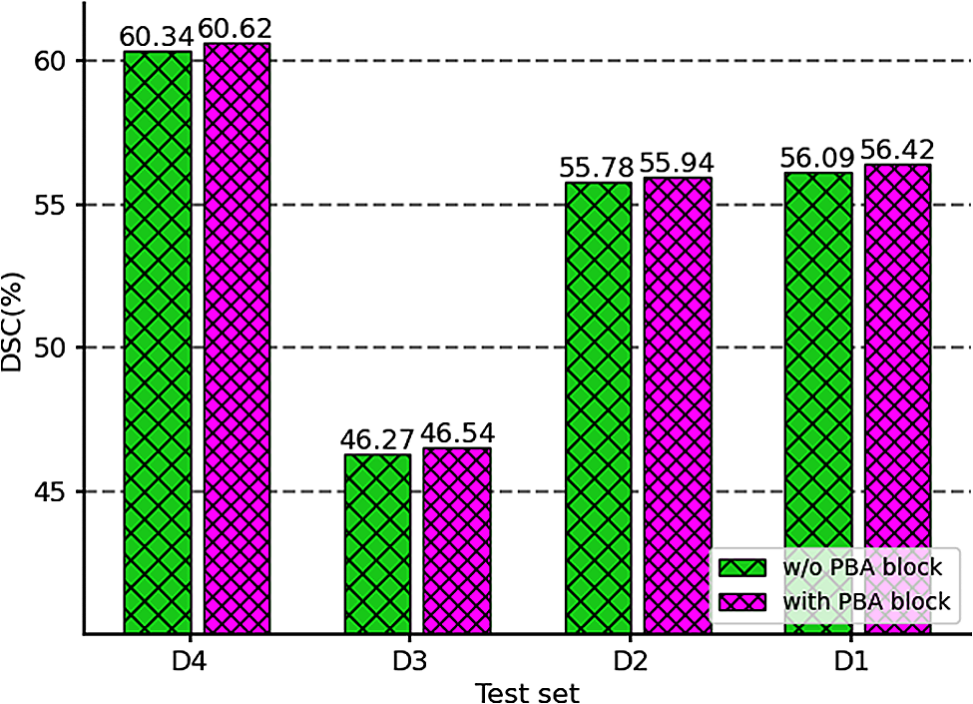
The DSC values obtained by the proposed AsTr in four cross-center test scenarios with or without the help of PBA block

In medical image segmentation task, it is very important to accurately draw the lesion/object boundary. As shown in Fig. 3, we use the bar graph to plot the segmentation results of AsTr with or without the PBA block. We can intuitively see that adding PBA blocks to the decoding path can further improve segmentation performance. Although the PBA module demonstrated weak performance gains, it was able to steadily refine prediction boundaries in the four cross-center test scenarios. In Fig. 4, we also visualized 2D axial views of some segmentation results, which not only showed that the prediction of lesion boundaries by the proposed method was closer to the ground-truth, but also confirmed that PBA blocks were helpful for boundary refinement.

#### Effectiveness of the HCADA module

The core of the module is to consider tumor size and class-specific information during feature alignment to improve segmentation performance of segmentation network AsTr in the cross-center test scenario. Therefore, we can consider only one of the above two factors to conduct the comparative experiment. Table 5 lists the segmentation results of these comparison methods. M1 means that only the last CADA block is used to complete the feature alignment, and M2 represents class-agnostic during feature alignment. According to the quantitative results, we believe that considering only one of the above two factors can also improve the segmentation performance, and considering both factors at the same time has the best performance.

**Table 5:** Segmentation results of different UDA methods in the cross-center test scenario

Training	Test	Method	DSC	JI	ASD	95HD
{D1, D2, D3}	D4	AsTr (Ours)	60.62	46.88	8.13	25.80
		M1	61.06	47.39	9.21	27.63
		M2	61.35	47.71	**7.28**	**21.06**
		HCA-DAN (Ours)	**62.20**	**47.98**	7.55	22.63
{D1, D2, D4}	D3	AsTr (Ours)	46.54	33.61	10.97	30.92
		M1	47.72	33.99	11.01	29.46
		M2	48.21	35.57	**10.15**	**28.80**
		HCA-DAN (Ours)	**49.25**	**36.86**	10.87	31.68
{D1, D3, D4}	D2	AsTr (Ours)	55.94	42.15	**10.50**	**4.52**
		M1	55.86	42.02	12.56	28.43
		M2	56.11	42.58	11.90	27.36
		HCA-DAN (Ours)	**56.72**	**43.39**	11.05	26.39
{D2, D3, D4}	D1	AsTr (Ours)	56.42	43.14	8.68	23.29
		M1	56.98	43.70	8.44	23.56
		M2	57.28	44.15	7.69	21.85
		HCA-DAN (Ours)	**58.36**	**45.18**	**6.91**	**20.64**

## Discussion

With the development of medical imaging equipment and deep learning algorithms, more and more deep learning-based methods are proposed for automated analysis of various cancers in various imaging modes. However, fully deep learning-based algorithms are still blank in the characterization and analysis of gastric cancer. In addition, there is heterogeneity among the multi-center data, which limits the deployment of the model in the clinic. To this end, we developed an intelligent analysis method for gastric cancer characterization and analysis in this paper, which not only achieves better segmentation performance by effectively bridging CNN and Transformer, but also realizes the cross-center test scenario via a new domain adaptive technology.

Our approach can automatically characterize gastric cancer and provide a whole tumor segmentation, which helps determine appropriate surgical approaches and predict prognosis. Although our approach outperforms other segmentation methods, there is still room for improvement in the tumor segmentation task. We believe that there may be two reasons. On the one hand, the voxel space distance of the data limits the segmentation performance. On the other hand, the small objective segmentation task is interfered by the background area. Therefore, our future research should not only focus on the heterogeneity between multi-center data, but also achieve higher tumor segmentation performance through two-stage modeling. The two-stage modeling strategy is more consistent with the clinical workflow, that is, the clinician first roughly determines the ROI and subsequently performs detailed lesion delineation.

In order to fully explore the performance of different models, we present number of FLOPs, parameters and averaged inference time of the models in Table 6. Number of FLOPs and inference time are calculated based on an input size of 28 × 256 × 256. The proposed AsTr is a relatively small model with 18.67 M parameters and 388.09G FLOPs. For comparison, other transformer-based methods such as CoTr, UNETR, and Swin-Unet have 41.27 M, 145.85 M and 102.81 M parameters and 670.62G, 2201.41G and 1582.56G FLOPs, respectively. AsTr shows comparable model complexity and is significantly better than similar models. CNN-based segmentation models of VNet, 3D FPN and nnUNet have 45.60 M, 7.83 M and 44.80 M parameters and 676.23G, 56.71G and 691.17G FLOPs, respectively. Compared to these methods, AsTr has the second lowest parameters and FLOPs. Similarly, AsTr has the second lowest averaged inference time after 3D FPN and is significantly faster than other models.

**Table 6:** Segmentation results of different segmentation methods in the cross-center test scenario

Training	Test	Method	DSC (%)	JI (%)	ASD	95HD
{D1, D2}	D4	V-Net	51.89	37.84	12.22	33.01
		3D FPN	52.05	38.02	10.98	32.20
		nnU-Net	53.96	40.15	9.48	**27.15**
		CoTr	52.54	38.09	6.39	27.29
		AsTr (Ours)	**57.80**	**43.86**	**4.32**	27.16
{D1, D4}	D2	V-Net	45.09	32.01	15.35	35.83
		3D FPN	44.95	31.86	15.62	36.03
		nnU-Net	46.48	33.20	10.84	35.80
		CoTr	46.34	33.00	14.42	42.05
		AsTr (Ours)	**53.10**	**39.28**	**8.51**	**24.28**
{D2, D4}	D1	V-Net	49.52	36.98	12.33	30.28
		3D FPN	50.68	37.79	10.95	29.33
		nnU-Net	51.01	38.25	**9.89**	**27.02**
		CoTr	51.93	38.80	10.03	28.34
		AsTr (Ours)	**53.44**	**40.67**	13.43	28.39

In addition, dataset D3 is particularly special in our four datasets. The voxel spacing between slices is 8 mm, which is different from the other three datasets. To explore this effect, we also set up a cross-center experiment without the participation of dataset D3. Table 7 lists the segmentation results of different segmentation methods. Comparing the segmentation results in Table 2, we found that the results decreased in all three cross-center test scenarios, indicating that the amount of data was more important than data quality in our cross-center gastric tumor segmentation scenarios. Therefore, we will collect and study more centers and data in the future.

**Table 7:** Comparison of number of parameters, FLOPs and averaged inference time for different models

Indicator	V-Net	3D FPN	nnUNet	CoTr	UNETR	Swin-Unet	AsTr
Param (M)	45.60	7.83	44.80	41.27	145.85	102.81	18.67
FLOPs (G)	676.23	56.71	691.17	670.62	2201.41	1582.56	388.09
Inference Time (s)	5.98	3.69	6.25	5.76	19.87	14.69	4.52

## Conclusions

In this paper, we propose a new HCA-DAN for cross-center 3D gastric tumor segmentation, which can not only learn discriminative multi-scale features from the CT images with anisotropic resolution, but also mitigate domain shift between cross-center datasets. In HCA-DAN, we first extract multi-scale features by combining anisotropic and isotropic convolution layers, and then employ DeTrans layers to model long-distance dependence in multi-scale features. Finally, we introduce the HCADA module to solve the problem of data distribution migration for better domain adaptation, in which we use the different size and class-specific information of the lesion in the 3D representation. The extensive experiments under four test scenarios together with comprehensive ablation study and analysis demonstrate the effectiveness of our approach for cross-center 3D gastric tumor segmentation.

Although domain adaptation technology can effectively handle domain shift, domain adaptation-based methods require images from the target domain (labeled or unlabeled) for real-time model training or retraining. In real-world scenarios, it is time-consuming or even impractical to collect data from each new target domain to fine-tune the model before deploying it. In future work, we will employ domain generalization technology to address the domain shift problem in multi-center study. The goal of domain generalization technology is to learn a model from a single or multiple source domains so that it can be directly generalized to unseen target domains, which facilitates the widespread use and effective deployment of intelligent analysis models in the clinic.

## Data Availability

The original contributions presented in the study are included in the article. Further inquiries can be directed to the corresponding author.

## Abbreviations

CT: Computed Tomography
CNNs: Convolutional Neural Networks
HCA-DAN: Hierarchical Class-Aware Domain Adaptive Network
AsTr: Anisotropic neural network and a Transformer
HCADA: Hierarchical Class-Aware Domain Alignment
ROI: Region of Interest
CAD: Computer-Aided Diagnosis
DQN: Deep Q Network
NLP: Natural Language Processing
CV: Computer Vision
UDA: Unsupervised Domain Adaptation
DANN: Domain Adversarial Neural Network
CyCADA: Cycle-Consistent Adversarial Domain Adaptation
SIFA: Synergistic Image and Feature Alignment
HCA-DAN: Hierarchical Class-Aware Domain Adaptive Network
PBA: Pyramid Boundary-aware
GN: Group Normalization
PReLU: Parametric Rectified Linear Unit
DSC: Dice Similarity Coefficient
JI: Jaccard Index
ASD: Average Surface Distance
